# Features and rhizosphere colonization strategies of *Lactobacillus plantarum* 0308 in soil-tomato systems

**DOI:** 10.3389/fmicb.2025.1652881

**Published:** 2025-08-29

**Authors:** Xiancui Zhang, Haoran Liao, Tong Cai, Peiwen Cai, Xiangyu Wu, Zhe Wang, Haoyu Ma, Guoqiang Qiu, Mingxing Zhao, Xingmeng Lu, Xianting Wang, Choufei Wu

**Affiliations:** ^1^School of Life Sciences, Huzhou University, Huzhou, China; ^2^Shandong Center for Disease Control and Prevention, Jinan, China; ^3^Deqing County Ecological Forestry Integrated Service Center, Huzhou, China; ^4^Laboratory of Invertebrate Pathology, College of Animal Sciences, Zhejiang University, Hangzhou, China; ^5^Hynobius Anji National Nature Reserve, The Administration Bureau of Longwangshan Natural Reserve, Anji, China

**Keywords:** colonization, *Lactobacillus plantarum*, microbial composition, rhizosphere soil, tomato

## Abstract

**Introduction:**

The lactic acid bacteria (LAB) has shown great potential as a sustainable solution to support agriculture through its plant-growth-promoting and biocontrol activities. However, their efficacy as bioinoculants is limited by unpredictable colonization in natural conditions.

**Methods:**

The bacterial strain LP0308, identified as *Lactobacillus plantarum* (LP0308) based on 16S rRNA sequence analysis, was obtained from rhizosphere soil. The features and colonization strategies of LP0308 were characterized through genome sequencing, tomato seed germination assays, pot experiments, and measurements of soil physicochemical properties and enzyme activities.

**Results:**

LP0308 was introduced into the soil of tomato, and it could stably persist and proliferate for a long-term (0–20 days), as confirmed by colony-forming unit (CFU), quantitative real-time PCR (RT-qPCR), and fluorescence *in situ* hybridization (FISH) analyses. Further characterization revealed that LP0308 altered the microbial composition of the rhizosphere soil and significantly increased the abundance of *Bacillus* and potentially pathogenic microorganism. Further analyses revealed that LP0308 altered the rhizosphere soil microbial community, significantly increasing the abundance of *Bacillus* spp. while decreasing the potential pathogenic microorganisms, such as *Ralstonia solanacearum* and *Fusarium oxysporum*. In addition, the successful colonization of LP0308 led to drastically increased expression of encoding biofilm (*vpsI*1, *vpsI*2, *vpsC*, and *vpsI*3), immune modulation (*pbpG*, *kdtB*, and *wbpL*), and antimicrobial activity gene (*farB*). *L. plantarum* strain LP0308 was confirmed as a possible plant growth-promoting rhizobacteria (PGPR), which significantly promoted bud length, plant height, primary root length, root fresh weight, and whole-seedling fresh weight. Additionally, application of LP0308 markedly improved soil nutrient availability and stimulated key enzymatic activities.

**Discussion:**

Together, our findings suggest the LP0308 as a potential target for developing more effective bioinoculants for sustainable agriculture.

## Introduction

Global agricultural production and food security have long been fundamental to human survival and development (Singh et al., 2023; Feng et al., 2025). Traditional agriculture often relies on chemical fertilizers and pesticides to boost yields (Carvajal-Yepes et al., 2019). However, by 2030, the global population is expected to reach 8.9 billion, and by 2050, it will grow to 9.86 billion, leading to an estimated 70% increase in the demand for food production (Kah et al., 2019). This increase will put immense pressure on agricultural output and land resources, which are already facing mounting challenges (van Vliet et al., 2017). Among these, accelerated urbanization, soil degradation, and the environmental pollution caused by the excessive use of pesticides and fertilizers are becoming more prominent (Borrelli et al., 2020; Tang et al., 2021). To address these challenges and achieve the United Nations Sustainable Development Goals (SDGs), there is an urgent need to develop alternative fertilizers that can replace traditional chemical fertilizers (Hofmann et al., 2020; McElwee et al., 2020).

Plant growth-promoting rhizobacteria (PGPR) are considered potential beneficial microorganisms capable of forming symbiotic relationships with plants (Wang et al., 2024). PGPR, such as *Bacillus*, *Klebsiella*, and *Pseudomonas* species, play a crucial role in enhancing plant health, improving soil fertility, boosting plant immunity, and facilitating the assimilation of essential nutrients (Wang, 2009; Jansson et al., 2023). However, the inconsistent or suboptimal effectiveness of microbial inoculants in agricultural soils over time may be attributed to the complex soil environment, microbial niche competition, and limited root colonization ability, which hinder the expression of biological activity factors by target strains (Britt, 2021; Khoso et al., 2024). In addition, microbial inoculants are subject to various environmental pressures, such as climate change and abiotic stressors, which can make them seem like “invaders” (Sammauria et al., 2020; Liu et al., 2022). They must also compete for ecological niches with indigenous microbial communities, further reducing their adaptability and limiting largescale use (Moore et al., 2022). Root zone colonization is a critical mechanism through which probiotics exert their beneficial effects, significantly influencing the efficacy of microbial agents utilized as biofertilizers, biopesticides, plant growth stimulants, and bioremediation agents (Knights et al., 2021; Liu et al., 2024). Rhizosphere colonization is one of the most important features of rhizobacteria that determines their survival and propagation, which are prerequisites for versatile bacteria to exert their beneficial functions on host plants (Santoyo et al., 2021). The process of colonization can be delineated into several key stages, including chemotaxis and motility, attachment to the root surface, overcoming plant immunity, proliferation and biofilm development on the rhizoplane, followed by endophytic penetration (Knights et al., 2021). For example, NCT-2 significantly enhanced nitrate removal from the soil and improved plant growth by colonizing the meristematic and elongation zones of the root tip as well as the roots (Chu et al., 2018).

Lactic acid bacteria (LAB), a group of Gram-positive microorganisms, exhibit symbiotic, biological defense, and environmental remediation properties, offering considerable potential for advancing sustainable agriculture and environmental protection (Ayivi et al., 2020; Rama et al., 2024). LAB strains, recognized for their probiotic properties, high safety, and broad applicability, have been granted Generally Recognized as Safe (GRAS) status by the U.S. Food and Drug Administration, making them an ideal candidate for commercial development in food, sustainable agriculture, and pharmaceutical industries (Hati et al., 2013; Sadiq et al., 2019). Studies have shown that LAB are widespread in the phyllosphere, endosphere, and rhizosphere of various plant species, such as maize, pepper, and barley. Furthermore, studies have found that LAB have garnered significant attention in sustainable agriculture due to their presence as components of the epiphytic and/or endophytic microbial communities in feed crops and roots. For example, when inoculated into *Lolium perenne*, *Lactobacilli* can colonize the roots at an endophytic level (Lamont et al., 2017). As a fertilizer, LAB can promote biodegradation, accelerate soil organic content, and produce organic acids and metabolic products such as plant growth-promoting (PGP) substances and nitrogen sources, helping plants improve nutrient absorption and stress resistance (Ekundayo, 2014; Minervini et al., 2015). For example, compared to untreated soil, the application of *Lactobacillus plantarum* H64 biomass combined with wheat bread significantly enhanced the soil organic carbon content (by up to 37%), total nitrogen content (by up to 40%), and the growth of *Cichorium endivia* in pots (1.7-fold increase) (Cacace et al., 2022). Additionally, LAB has shown antagonistic effects against plant pathogens, even inhibiting pathogenic fungal and bacterial populations in the rhizosphere and phyllosphere (Daranas et al., 2019; Chen et al., 2023). Moreover, recent research has found that LAB exhibits bioremediation efficiency and the ability to detoxify heavy metals and mycotoxins (Kirillova et al., 2017; Kinoshita, 2019). The application of LAB for heavy metal bioremediation in leafy vegetables demonstrated maximum adsorption efficiencies of 79.75% for lead (Pb), 75.28% for cadmium (Cd), and 83.99% for nickel (Ni) (Mostafidi et al., 2023).

Rhizospheric microorganism represent the first line of defense in maintaining soil health and protecting against soil-borne diseases. For example, *Burkholderia cenocepacia* strain XXVI produces hydroxamate siderophore with biocontrol activity against the pathogen *Colletotrichum lindemuthianum* ATCC MYA 456, promoting plant growth (Santos-Villalobos et al., 2012; Santoyo et al., 2021). However, limited research has been conducted on the colonization mechanisms of LAB in the rhizosphere and their PGP properties (O’Banion et al., 2023; Sanchez-Gil et al., 2023). In the current study, a LP0308 strain was isolated and purified from the rhizosphere soil of healthy tomatoes, and its characteristics were analyzed through whole genome sequencing. We further investigated the colonization of LP0308 in rhizosphere soil and its effect on rhizosphere microbial community structure of tomato root. In addition, we quantified colonization-related genes following the growth of the bacterium in the soil using quantitative real-time PCR (RT-qPCR). Finally, we analyzed indicators of plant growth promotion, soil physical and chemical properties, and enzyme activity following inoculation with LP0308. The present study enhances our understanding of rhizosphere soil colonization by an indigenous bacterium and may promote the application of probiotic targeting regulation as biofertilizers in agricultural.

## Materials and methods

### Isolation and identification of LP0308

Rhizosphere soils were collected as described previously (McPherson et al., 2018). Briefly, plants were gently handled to remove loosely adherent soil from their roots, while deliberately retaining an approximately 1-mm-thick layer of soil on the root surfaces (*n* = 15). Tomato rhizosphere soil was harvested in a tomato field in Zhejiang province, China (120.13°N, 30.87°W). LP0308 strain were isolated from the rhizosphere soil and purified by plate culture with de Man–Rogosa–Sharpe medium (MRS) medium (Hopebio, Qingdao, China). Bacterial colonies were randomly selected from agar plates and subcultured a minimum of three times prior to identification. The growth curve of LP0308 was determined using 96-well honeycomb plates (Corning, United States), with optical density measured at 600 nm at 5 h intervals over a 50 h period.

For scanning electron microscopy (SEM) analysis, LP0308 was initially fixed in 2.5% glutaraldehyde, washed three times with PBS, and subsequently post-fixed in 1% osmium tetroxide. The samples were then dehydrated through a graded ethanol series (50%, 70%, 80%, 95%, and 100%) for 15 min and embedded in epoxy resin (Dr. Spurr’s kit, Electron Microscopy Sciences, Hatfield, PA, United States). Ultrathin sections were stained with 1% uranyl acetate and examined using SEM transmission electron microscope (Hitachi SU8000, Tokyo, Japan) at an accelerating voltage of 80 kV.

### *L. plantarum* stability and proliferation in the soil

The tomato varieties Sweetheart No. 8 were selected for their pomological traits which are highly appreciated by consumers of china. Seeds (*n* = 100 per variety) were provided by Yinong Agriculture Co., Ltd. (Zhejiang, China). For the seed germination assay, surface-sterile tomato seeds prepared as follows. They were surface-sterilized by immersion in 70% ethanol (Sangon, Shanghai, China) for 1 min, followed by treatment with 10% hypochlorite (Sangon, Shanghai, China) for 5 min, and then subjected to five consecutive rinses with sterile distilled water. For the root colonization assay, surface-sterile tomato seeds prepared as described above. Tomato seedlings at the two- to three-leaf stage, grown under sterile conditions, were immersed in LP0308 bacterial suspension (106 CFU/L) for 4 h before being transplanted into sterile soil and incubated in a climate-controlled sterile growth chamber (Percival growth chambers; CLF Plant Climatics) of 22 °C/18 °C under a 16 h/8 h (day/night), with blank medium (CK) as the control. At 5-day intervals, rhizosphere soil tightly adhering to the root surface was carefully collected and serially diluted with sterile water. All compartments were placed on ice. The number of *L. plantarum* in rhizosphere soil was quantified by standard colony-forming unit (CFU) enumeration and absolute quantification via RT-qPCR after inoculation with LP0308 5, 10, 15, and 20 days later. CFU counts were determined by serial dilution of each sample (1 × 10^–1^ to 1 × 10^−6^), plating, and incubation at 30 °C for 48 h. Colonies were then manually counted (*n* = 5). The experiment was repeated five times. The colony-forming units per gram of soil (CFU/g) were then enumerated to assess bacterial colonization dynamics. For qPCR, the rhizosphere soil was collected and transferred to PowerBead Tubes (QIAGEN DNeasy PowerSoil Kit, Hilden, Germany) for LP0308 counts analysis. PowerBead Tubes containing soil samples were homogenized in a Precellys-24 tissue Homogenizer (Bertin Technologies, Aixen, France) at 5,000 rpm for 45 s. Total DNA was extracted using QIAGEN Dneasy PowerSoil Kit for soil according to the manufacturer’s instructions. The final DNA concentration and purity were determined by NanoDrop 2000 UV-vis spectrophotometer (Thermo Scientific, Wilmington, NC, United States), and DNA quality was checked by 1% agarose gel electrophoresis.

Quantitative PCR was performed by Roche LightCycler 480 system (Roche, Basel, Switzerland) using the SYBR qPCR master mix (Vazyme Biotech, Nanjing, China). The primer sequences for all amplified genes are shown in Supplementary Table 1: LP for LP0308 and BA1 for *Bacillus* (Chen et al., 2010; Bodenhausen et al., 2014; Roosa et al., 2014). RT-qPCR was conducted using the following program: 5 min of denaturation at 95 °C, followed by 40 cycles of 10 s at 95 °C, 10 s of annealing at an appropriate temperature, and 10 s of elongation at 72 °C, followed by a final melting curve step (from 65 °C to 92°C, 0.5 °C/s). The copies were calculated by comparing Cp values to a standard curve amplified from a series of 10-fold dilutions of containing purified genomic DNA for individual gene quantification. The same approach to confirm the accuracy of *Bacillus* copies number. Each group included at least three technical replicates and five biological replicates.

### Fluorescence *in situ* hybridization analysis of LP0308 in soil

Fluorescence *in situ* hybridization (FISH) analysis of LP0308 in rhizosphere soil. LP0308 was fixed with FISH fixative for 30 min at room temperature, permeabilized in PBS with 0.2% Triton X-100 for 5 min, and then incubated in pre-hybridization solution at 37 °C for 1 h. The fixed cells were then hybridized with the fluorescently probe LbpV3 (5′-CCGTCAATACCTGAACAG-3′) lactobacillus–specific probe overnight at 4 °C (Gosiewski et al., 2012). Then, the cells were washed five times with PBS and stained with 1:1,000 DAPI solution for 5 min. Fluorescence images were captured using a fluorescence microscope (CryoStar, NX70, France).

### The rhizosphere soil microbiota analysis by 16S rRNA gene sequencing

Two rhizosphere soil samples (CK and LP0308 group, 5 g each) per plant were collected and immediately processed for DNA extraction. For each plant, DNA was extracted from 0.3 g of rhizosphere soil using the DNeasy PowerSoil kit (Qiagen, Hilden, Germany) according to the manufacturer’s instructions. To construct an Illumina sequencing library, the V4–V5 hypervariable regions of the 16S rRNA gene were amplified from 1 μl of purified DNA using the universal primers 515F and 806R, as listed in Supplementary Table 1. Purified PCR productions were pooled in equimolar and paired-end sequenced (2 × 300) on an Illumina MiSeq platform (Illumina, San Diego, CS, United States) following the standard protocols provided by Majorbio Bio-Pharm Technology Co., Ltd. (Shanghai, China). Twenty bacterial samples were successfully sequenced.

To investigate the rhizosphere soil bacterial community structure, alpha-diversity indices (Shannon and Simpson indices; the number of observed species) were calculated using Mothur. Bray–Curtis distances between samples were used for principal coordinate analysis (PCoA, cmdscale function in R). To test the effect of location and compartment on the estimated explained variance, a permutational multivariate analysis of variance (PERMANOVA) analysis was performed (Adonis function from the vegan package, in R). Network analyses were used to explore the co-occurrence patterns of rhizosphere soil bacterial microorganisms in each niche. Moreover, linear discriminant analysis (LDA) effect size (LEfSe) was conducted using the normalized ASV table.

To assess the difference in rhizosphere microorganisms between the CK and LP0308 colonization groups (LP), the molecular ecological networks among the top 100 genera with the highest abundance of bacterial taxa were described through network analysis using the Molecular Ecological Network Analyses Pipeline (MENAP) (Deng et al., 2012). The correlation matrix was calculated by the Pearson correlation coefficient. Networks were constructed using random matrix theory-based methods with a correlation threshold of 0.65 (Zhou et al., 2010; Zhou et al., 2011). The resulting correlations were imported into the Gephi platform and then visualized by the Fruchterman Reingold algorithms (Bastian et al., 2009). The topological features of every node, including clustering, average degree, degree, and closeness centrality, were calculated for each graph (Shannon et al., 2003).

Finally, phylogenetic investigation of communities by Functional Annotation of Prokaryotic Taxa (FAPROTAX) was employed for stringent predictions of microbial functional metabolic pathways from the Kyoto Encyclopedia of Genes and Genomes (KEGG) pathway enrichment analysis. The phenotypes of rhizosphere soil bacteria in each group (CK and LP0308) were predicted using the BugBase database. Sequencing data were deposited in the National Center for Biotechnology Information (NCBI) Short Read Archive (SRA) under accession number PRJNA1270370.

### Antagonistic effect of LP0308 against soil borne pathogens

To evaluate the antagonistic activity of LP0308 against common soil-borne pathogens of tomato, five phytopathogenic strains were selected from laboratory collection: *Ralstonia solanacearum*, *Fusarium oxysporum*, *Cladosporium fulvum*, *Alternaria solani*, and *Botrytis cinerea*. Uninoculated liquid medium and sterile water were used as a negative control. The LP0308 strain was cultured in MRS medium at 37 °C for 24 h. The supernatant was collected after centrifugation at 10,000 × *g* for 3 min and filtered through a 0.22 mm PVDF membrane (Millipore Corp., Billerica, MA, United States) to obtain the cell free fermentation liquid. Antimicrobial activity of LP0308 was evaluated using the agar well diffusion method (Zhang et al., 2022).

### Germination of tomato seeds and pot experiment

The seeds were treated with sterile LP0308 fermentation supernatant. Bud length was measured with a ruler for plants in the CK (sterile MRS liquid medium) and LP0308 (LP) groups. Germinated seedlings were transferred into 10 cm × 8.5 cm pot with each pot containing one seedling. Each seedling pot was filled with approximately 150 g of sterilized soil. After 20 days, plant height and root length were measured with ruler. The root fresh weight and seedling fresh weight were measured with a precision balance. Then, the 0.2 g fresh root sample was extracted with 2 ml of phosphate buffer (pH 7.4) and then centrifuged at 1,000 *g* for 10 min. The contents of indoleacetic acid (IAA) of tomato root were measured via enzyme-linked immunosorbent assay (ELISA) according to the protocols (ELISA kits BH100, Zoonbio Biotechnology, Nanjing, China). The experiment was repeated 5 times, each time with 15 tomato plants.

### Determination of soil physicochemical properties and enzyme activities

Soil samples were collected using a sterile dry brush to gently scrape the surface soil from the roots of control and LP0308-treated tomato plants. Soil pH was measured using a pH meter on an appropriate amount of air-dried soil (sieved through a 20-mesh sieve), which was suspended in a 1:2.5 soil-to-water ratio. The soil organic matter (OM) was measured using the wet oxidation redox titration. Ammonium and nitrate were analyzed by a continuous-flow analyzer. The available phosphorus (AP) was measured using the molybdenum blue method. In addition, we measured the alkaline nitrogen (AN), and available potassium (AK), following the method (Bao, 2000; Nichols and Wright, 2006). The activities of several soil enzymes were assessed, including polyphenol oxidase (POX), urease, β-glutaminase (GLS), peroxidase (POD), alkaline phosphatase (ALP), cellulase (CE), N-acetyl-β-D-glucosaminidase (NAG), and β-1,4-glucosidase (BG). All soil enzyme activities were measured with a commercial activity assay kit (Solebao, Beijing, China) according to the manufacturer’s instructions.

### Genome sequencing, assembly, and annotation

The whole genome of LP0308 was sequenced using the PacBio RSII platform at Majorbio Bio-Pharm Technology Co., Ltd. (Shanghai, China), generating 2,266 Mbp of high-quality reads. To ensure data integrity, sequences with non-standard bases at the 5′ ends, lengths under 25 bp, more than 10% ambiguous bases (Ns), or a quality score ≤ 20 were filtered out. Genome assembly was conducted using SOAPdenovo v1.05 with various k-mer sizes, and remaining gaps were closed through PCR amplification (Liang et al., 2018; Xie et al., 2019). The raw sequencing data, including both single-end and paired-end reads, are available in GenBank under BioProject ID PRJNA1269284. Gene prediction for strain LP0308 was carried out using Glimmer v3.02.

Genes of strain LP0308 prediction was performed using Glimmer, while tRNA and rRNA genes were identified with tRNA-scan-SE and Barrnap, respectively. The predicted CDSs were then annotated by aligning sequences against multiple databases, including NR, Swiss-Prot, Pfam, GO, COG, and KEGG, utilizing tools such as BLAST, Diamond, and HMMER. In brief, query proteins were compared to these databases, and gene annotations were assigned based on the best matches with an *e*-value threshold of less than 105 (Ashburner et al., 2000). The raw dataset including both single and paired-end reads, is deposited at GenBank under BioProject and accession number ID: PRJNA763083.

### Quantitative real-time PCR validation of genes

After inoculating the soil with LP0308 (10^7^ CFU/ml), the expression levels of colonization-related genes [exopolysaccharide biosynthesis glycosyltransferase VpsI (gene1112, vpsI1), D-alanyl-D-alanine endopeptidase PBP7/8 (gene1112, pbpG), exopolysaccharide biosynthesis glycosyltransferase VpsI (vpsI2), exopolysaccharide biosynthesis acetyltransferase VpsC (vpsC), exopolysaccharide biosynthesis glycosyltransferase VpsI (vpsI3), lipopolysaccharide core biosynthesis protein (kdtB), glycosyltransferase WbpL (wbpL) and fatty acid efflux system protein FarB (farB)] were determined at 0, 5, 10, 15, and 20 days using RT-qPCR (*n* = 5).

Total RNA from the rhizosphere soil was extracted using the RNeasy^®^ PowerSoil^®^ Total RNA Kit (Qiagen, Hilden, Germany), following the manufacturer’s protocol. The RNA quality and concentration were measured with a Qubit Fluorometer (Thermo Fisher, United States). RNA of the desired quality (1 mg) was reverse transcribed into cDNA using QuantiTect Reverse Transcription Kit (Qiagen, Hilden, Germany). The specific primers used for RT-qPCR of colonization-related genes are listed in Supplementary Table 1. RT-qPCR was performed according to a previously described of this study. Gene expression was calculated relative to the housekeeping gene 23S rRNA using the 2^–ΔΔCt^ method.

### Statistical analysis

Statistical analyses were conducted using GraphPad Prism (version 9.0). Following assessment of data normality and homogeneity of variance, appropriate statistical tests (parametric or non-parametric) were selected. One-way analysis of variance (ANOVA) followed by Tukey’s *post-hoc* test or Student’s *t*-test was performed to compare the quantification of soil bacteria, bugbase predictions, topological features of network analysis, IAA contents, plant growth indicators, soil physical properties, and enzyme activities and gene expression between the experimental and control groups were analyzed. A *p*-value of <0.05 was considered statistically significant. All experiments were conducted with a minimum of three independent replicates.

## Results

### General features of the LP0308

**FIGURE 1:**
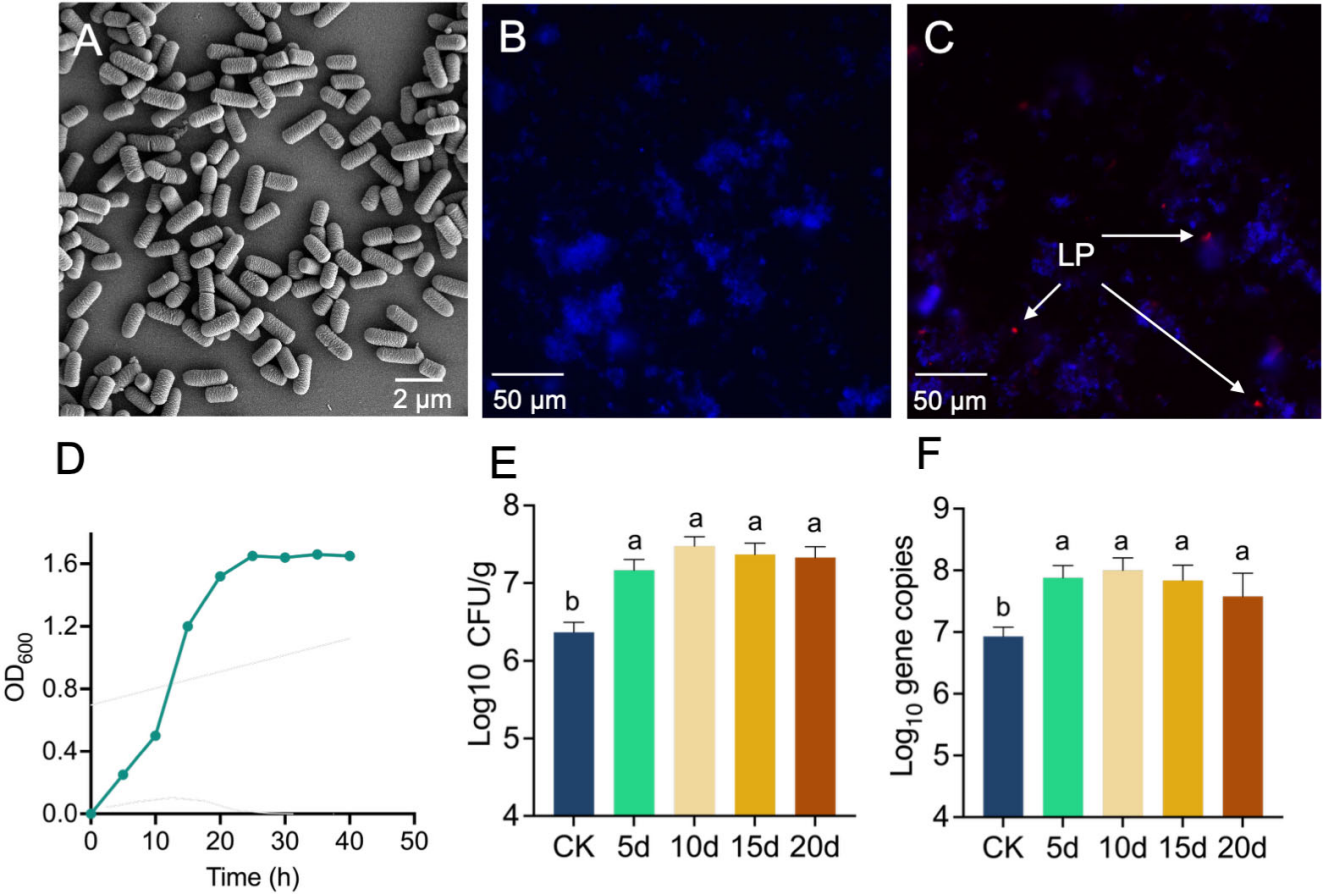
Characteristics and colonization of LP0308 in the tomato rhizosphere soil. **(A)** Scanning electron microscopy (SEM) images of LP0308. Scale bars are 2 μm. **(B)** Compared to the rhizosphere soil of normal group, FISH with a Cy3–labeled *Lactobacillus plantarum*-specific probe (red) showed large amounts of *L. plantarum* colonizing to the rhizosphere soil (blue) **(C)**. **(D)** The growth curve of LP0308 exhibits a typical S-shaped pattern. LP0308 colonizing the rhizosphere was quantified by CFU **(E)** and RT-qPCR **(F)**. Statistically significant changes were determined by ANOVA and the *post hoc* Tukey honestly significant difference (HSD) test. Different letters represent statistically significant differences (*P* < 0.05, *n* = 5).

A phylogenetic tree was constructed using whole-genome sequences to elucidate relationships among LAB strains. It showed that strain LP0308 was closely related to *L. plantarum* and was therefore designated LP0308 (Supplementary Figure 1). Electron microscopy revealed the bacterium was rod shaped and 1.5–2 μm in length, with a diameter of 0.6–1 μm (Figure 1A). FISH with a Cy3-labeled LP0308-specific probe (red) showed large amounts of *L. plantarum* colonizing to the rhizosphere soil (blue), compared to the rhizosphere soil of normal group (Figures 1B, C). The growth curve exhibits a typical S-shaped pattern. The strain remains in the lag phase for the first 10 h post-inoculation, enters the exponential phase from 10 to 20 h, and reaches the late logarithmic phase at 25 h (Figure 1D).

The LP0308 strain colonizing the rhizosphere was quantified by CFU and RT-qPCR (Figures 1E, F). Over time, there was a statistically significant increase in bacterial levels in the rhizosphere soil. The percentage of LP0308 bacterial increased from 2.32 × 10^6^ CFU/g to 3.0 × 10^7^ CFU/g during this period (0–10 days) (*t* = 6.302, df = 8, *P* = 0.0002). The density of strain LP0308 colonizing rhizosphere soil also reached 2.147 × 10^7^ CFU/g soil in 20 days, showing the long-term stability of LP0308 (Figure 1F). Independent experimental replicates, performed using RT-qPCR with universal 16S rRNA gene primers, yielded consistent results across different time points (Figure 1F).

### Effect of LP0308 on rhizosphere soil microorganisms

**FIGURE 2:**
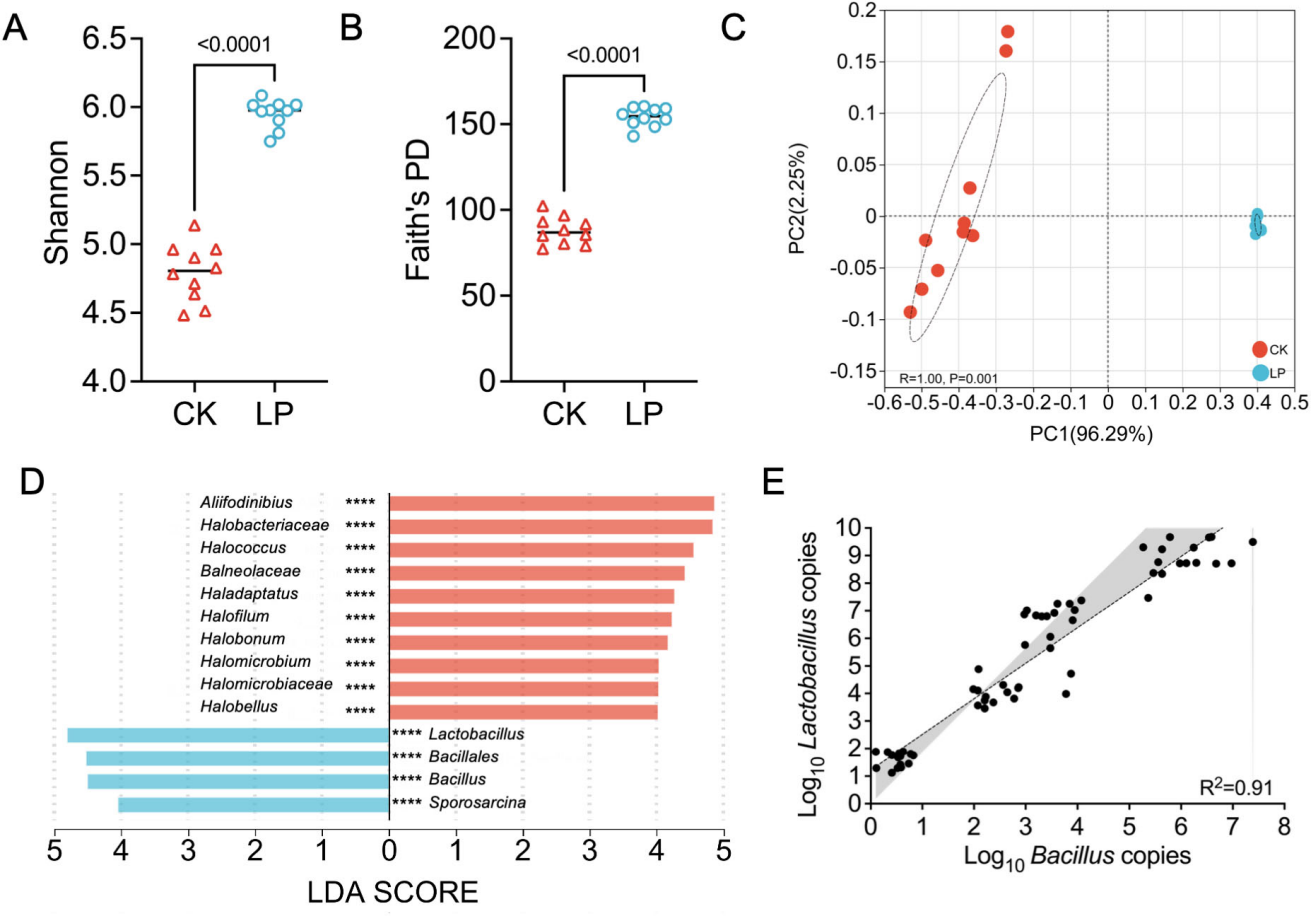
Colonization of LP0308 changed the composition of rhizosphere soil bacteria. **(A,B)** Alpha diversity and **(C)** beta diversity were investigated. Alpha diversity (Shannon and Faith’s PD) of bacterial communities (based on ASVs) at CK and LP0308 group (10 days). PCoA plot showing variation in community structure among CK and LP0308 group [permutational multivariate analysis of variance (PERMANOVA) test with 999 permutations, *P* > 0.05] based on Bray–Curtis distance. Each point represents an individual sample. **(D)** LDA effect size (Lefse) analysis was conducted to investigate the variations in rhizosphere soil microbial composition between the two groups (CK and LP0308). **(E)** Correlation between *Lactobacillus plantarum* and *Bacillus* at rhizosphere soil after inoculation with LP0308. Dotted lines denote the linear regressions. Statistically significant differences were calculated by using unpaired Student’s *t*-test (*P* < 0.05). **** indicates *P* < 0.0001.

We observed 885 unique bacterial ASV (CK) and 1,678 unique ASV (LP) on collected samples, respectively. Alpha diversity (Shannon index, Faith’s PD) indices indicated a gradual increase of microbial diversity from the CK to the LP group (Figures 2A, B). We found that the bacterial communities were divided into two main clusters along with the first PCoA axis according to the compartment, whereas the major factor explaining communities was the inoculation of LP0308. The two groups were significantly different (*P* = 0.001, PERMANOVA), and were treated differently as the particular spatial scales (Figures 2B, C and Supplementary Figures 2A, B). Lefse analysis was performed to explain the differences between rhizosphere soil microorganism composition between the two groups. Based on LDA effect size, *Lactobacillus*, *Bacillales*, *Bacillus*, and *Sporosarcina* were significantly enriched in LP0308 group, while *Aliifodinibius*, *Halobacteriaceae*, *Halococcus*, *Balneolaceae*, and *Haladaptatus* were mostly related to the uninoculated plants individuals (Figure 2D). The total number of *Lactobacillus* and *Bacillus* were estimated using DNA copy numbers measured by RT-qPCR (Figure 2E). Moreover, there was a statistically significant positive correlation between the total *Lactobacillus* number and the *Bacillus* number in the rhizosphere soil (*R*^2^ = 0.91) (Figure 2E).

### Assembly processes and coexistence in the rhizosphere soil microorganisms

**FIGURE 3:**
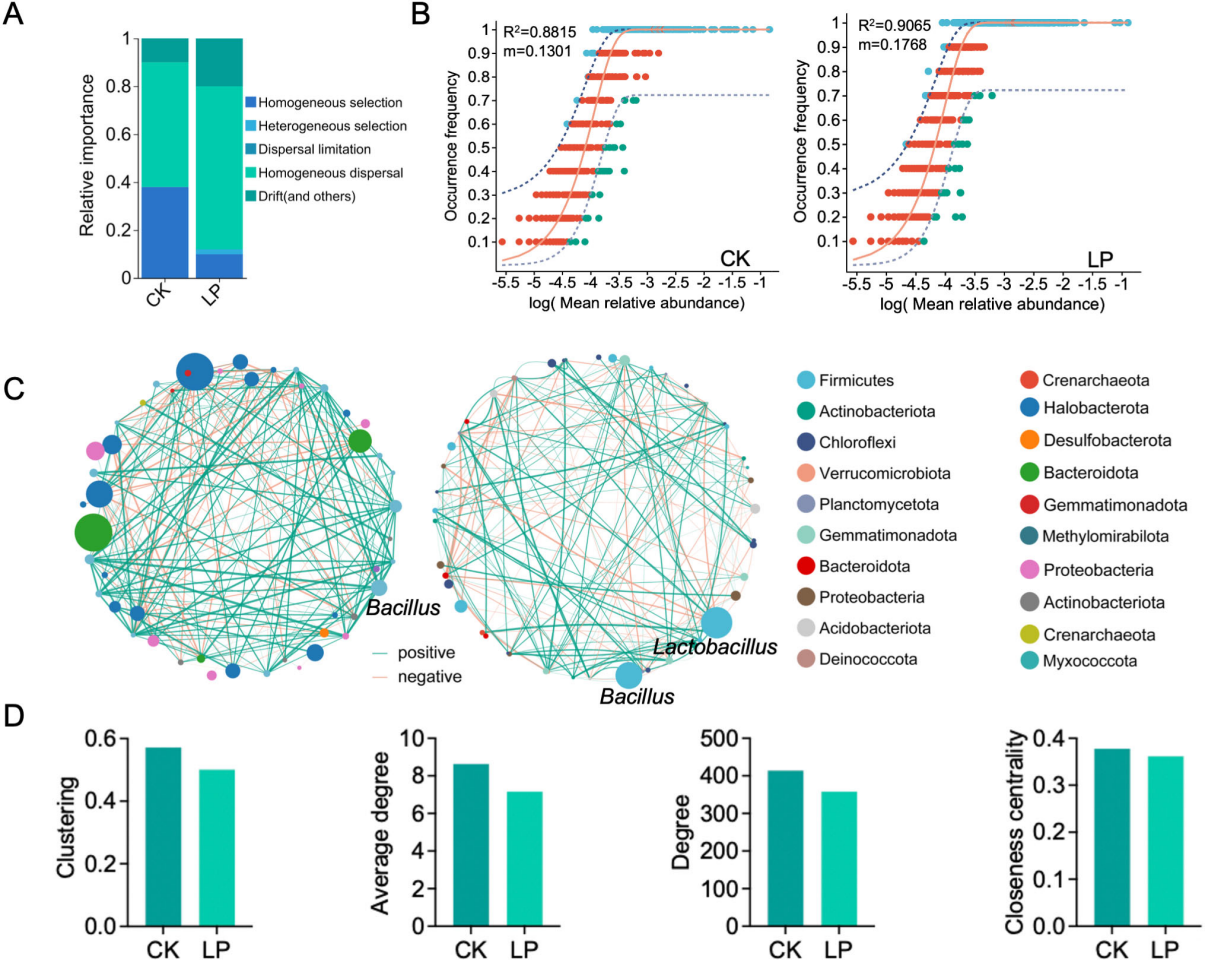
Community assembly and microbial network in rhizosphere soil bacteria mediated by *Lactobacillus plantarum* 0308. **(A)** Community-level drivers of rhizosphere soil-associated microbial communities assembly over study regions. The four components were described with to classify community pairs into underlying drivers “Selection” implying environmental filtering; “Homogenizing Dispersal” representing strong dispersal; “Dispersal limitation” suggesting weak dispersal; and “Drift” indicating stochasticity (“undominated”). **(B)** Fit of the neutral community model (NCM) of community assembly. The predicted occurrence frequencies for CK and LP0308 group representing bacteria communities, respectively. The dark purple dotted lines indicate the best fit to the NCM and the light purple dotted lines represent 95% confidence intervals around the model prediction. ASVs that occur more or less frequently than predicted by the NCM are shown in different colors. *Nm* indicates the metacommunity size times immigration, *R*^2^ indicates the fit to this model. **(C)** The correlation-based network of rhizosphere soil-associated microbial genus was detected in two different group (CK and LP0308). Each node corresponds to a genus, and edges between nodes correspond to either positive (green) or negative (red) correlations inferred from OUT abundance profiles using the random matrix theory-based methods with a correlation threshold of 0.65 in MENA. The size of each node is proportional to the abundance of the genus. *Lactobacillus* and *Bacillus* had the highest abundance in the LP0308 group, respectively. **(D)** Unique node-level topological features of different taxa categories, including clustering, average degree, degree, and the closeness centrality.

We then conducted neutral and null model analyses to quantitatively decipher the relative contribution of ecological processes including homogeneous selection (HoS), heterogeneous selection (HeS), dispersal limitation (DL), homogenizing dispersal (HD), and drift in driving the assembly of rhizosphere soil-associated bacterial communities (Figure 3A). Null model analysis showed that the assembly of normal group (CK) was dominantly shaped by HoS (38%), followed by HD (52%), drift and non-dominant processes (10%). However, the rare subcommunity assembly was shaped almost equally by deterministic (10% of HoS and 2% of HeS) and stochastic (68% of HD, 20% of drift and non-dominant) processes (Figure 3A). The two sample treatments (CK vs. LP) exhibit different βNTI patterns. βNTI values were significantly higher for bacterial communities of LP than LP0308 treatments (*P* < 0.05) (Supplementary Figure 2C). Finally, the neutral community model (NCM) successfully estimated a large fraction of the relationship between the occurrence frequency of ASV and their relative abundance variations, with 88.15% and 90.65% of explained community variance for CK and LP group, respectively (Figure 3B). The explained variation of NCM remained fairly consistent across taxonomic ranks from phylum to genus, suggesting that taxa within the same phylogenetic lineage generally respond similarly to stochastic processes. The *m* value was estimated to be 0.18 in LP group and 0.13 in CK, respectively. These results indicated that species dispersal of rhizosphere soil microorganism was higher after colonization of LP0308 (Figure 3B).

Subsequently, the constructed phylogenetic molecular ecological networks (pMENs) consisted of 414 edges and 358 edges from genera in the top 50 abundance in 60 samples. Interestingly, we observed that, in addition to the successful colonization of *Lactobacillus*, the abundance of *Bacillus* increased significantly compared to the control group (Figure 3C). The number of clustering (0.57 vs. 0.50), average degree (8.63 vs. 7.16), degree (414 vs. 358), and the closeness centrality (0.38 vs. 0.36) of the rhizosphere soil microorganism network (CK) were also considerably higher than for the treatment group network (Figure 3D). These finding, together with the network global properties, indicated that the CK group exhibited much closer interconnections than the treatment group at the phylum level.

**FIGURE 4:**
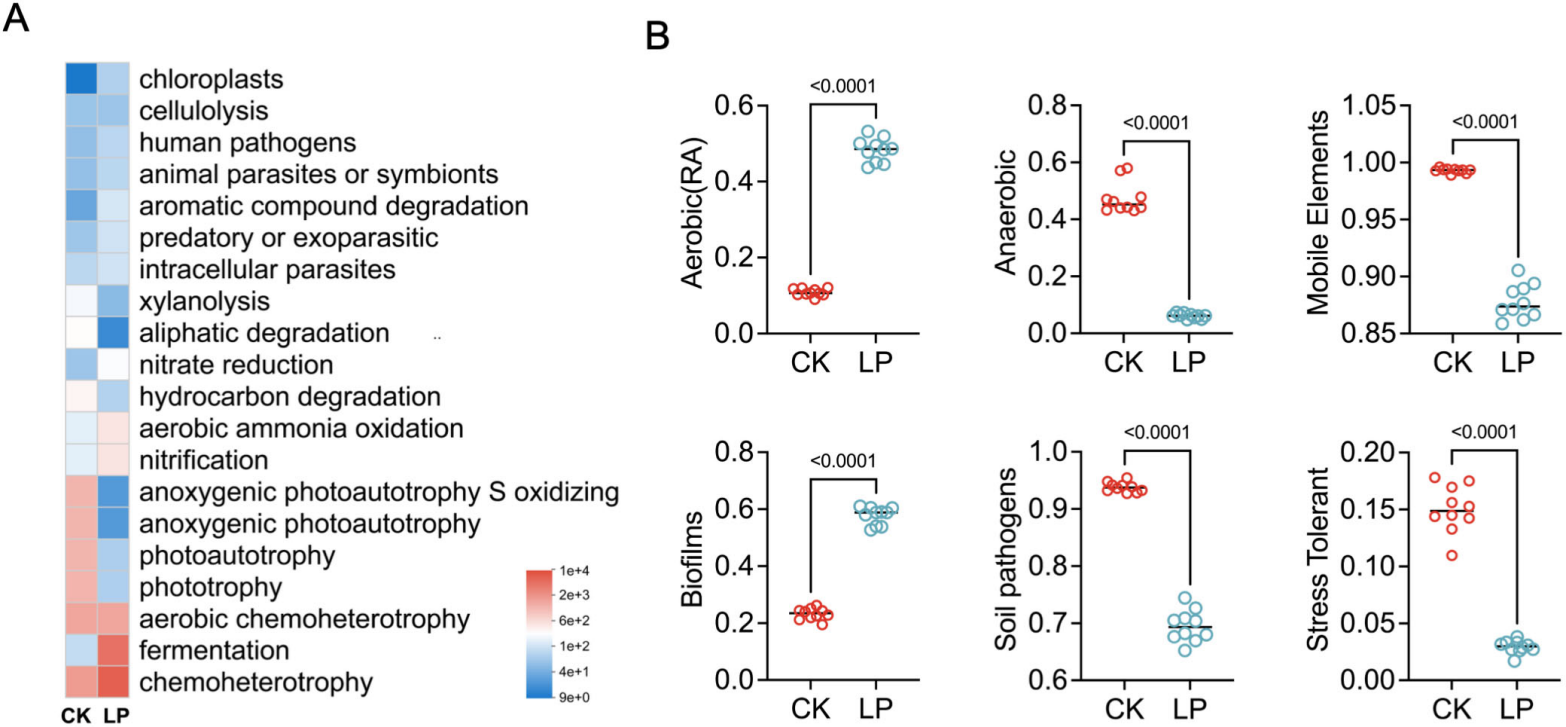
The functional content of the rhizosphere soil-associated microbial communities. **(A)** The heatmap result showed that the dominant functional groups by using FAPROTAX based on the baseline and endpoint 16S amplicon sequencing data between the CK and LP0308 treatment groups. **(B)** The phenotypes of rhizosphere soil bacteria in each group (CK and LP0308) were predicted using the BugBase database. Statistically significant differences were calculated by using unpaired Student’s *t*-test (*P* < 0.05).

In addition, functional profiles of rhizosphere soil bacterial community were predicted using FAPROTAX analyses, and significant differences among all groups were determined (Figure 4A). The heatmap result showed that the dominant functional groups were chemoheterotrophy (18.96% ± 2.82% vs. 40.90% ± 1.45%, *P* < 0.001), fermentation (1.09% ± 0.69% vs. 29.31% ± 2.54%, *P* < 0.001), aerobic chemoheterotrophy (15.42% ± 1.96% vs. 10.75% ± 1.42%, *P* < 0.001), phototrophy (11.52% ± 1.52% vs. 0.53% ± 0.09%, *P* < 0.001), and photoautotrophy (11.43% ± 1.52% vs. 0.53% ± 0.08%, *P* < 0.001) between the CK and LP0308 treatment groups (Figure 4A).

Phenotypes of the rhizosphere soil bacterial in each group were predicted based on the BugBase database. Interestingly, the sum of anaerobic (6.12% ± 0.83% vs. 47.49% ± 5.54%), mobile elements (87.81% ± 1.51% vs. 99.30% ± 0.20%), potentially pathogenic (69.50% ± 2.80% vs. 93.81% ± 0.87%), and stress tolerant (2.94% ± 0.57% vs. 15.05% ± 2.07%) were particularly lower in the LP0308-statins group than the control group (*P* < 0.001) (Figure 4B). However, a higher proportion of aerobic (48.30% ± 3.12% vs. 10.88% ± 0.98%) and a stronger potential to synthesize biofilms (57.73% ± 3.05% vs. 23.24% ± 2.02%) in the LP were observed when compared with the CK (*P* < 0.001) (Figure 4B).

Then, five representative soil-borne tomato pathogens were used to assess antagonistic effect of LP0308. *R. solanacearum* and *F. oxysporum* was inhibited by the LP0308 cell-free supernatant in the agar well diffusion assay; no growth suppression was detected for *C. fulvum, B. cinerea*, or *A. solani* (Supplementary Figure 3).

### LP0308 genomic features and rhizosphere colonization genes

**FIGURE 5:**
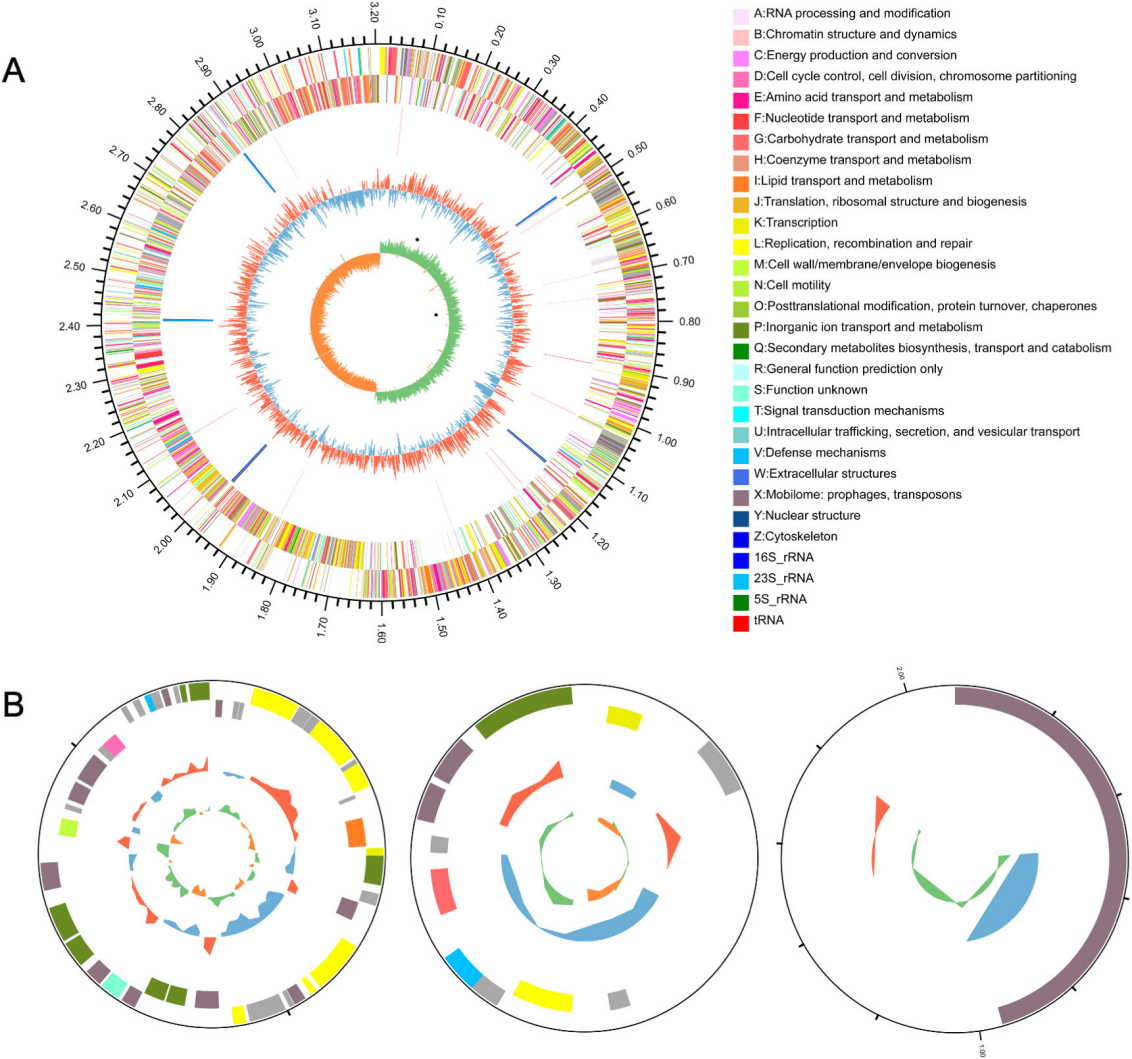
LP0308 strain genome resources and characteristics. Circular representation of the chromosome **(A)** and plasmid **(B)** of *Lactobacillus plantarum* strain LP0308. The outermost circle represents the total genome size. The second and third circles display sequences encoding protein amino acids on the + and– strands, with different colors indicating distinct COG functional classifications. The fourth circle denotes rRNA and tRNA. The fifth circle illustrates GC content, where outward red segments indicate regions with GC content higher than the genome-wide average, while inward blue segments indicate regions with lower GC content; the peak height reflects the magnitude of deviation from the average GC content. The sixth circle represents the GC skew value (G – C/G + C), where a positive value suggests a higher likelihood of CDS transcription from the positive strand, whereas a negative value indicates transcription from the negative strand.

LP0308 possesses a chromosome and three plasmids (Figure 5). The chromosome genome spans 3,207,787 base pairs and harbors 3,060 total genes. Of these, 2,391 and 2,137 genes are annotated in the COG and KEGG databases, respectively. The complete genome contains 2,980 protein-encoding sequences, 64 tRNA genes, 16 rRNA genes, and a CRISPR region. In addition, three circular plasmids, respectively, with the size of 245,534 and 89,508 base pairs were found (Table 1).

**TABLE 1:** Genome features of LP0308.

Attribute	Chromosome	Plasmid A	Plasmid B	Plasmid C
Size (bp)	3,207,787	46,691	9,254	2,094
G + C content (%)	44.66	38.47	37.35	38.3
Total genes	3,060	46	11	0
Protein coding genes	2,980	46	11	0
tRNA genes	64	0	0	0
rRNA genes	16	0	0	0
Genes assigned to COGs	2,391	31	7	1
Genes assigned to KEGG	2,137	28	6	0
CRISPR repeats	1	0	0	0

**FIGURE 6:**
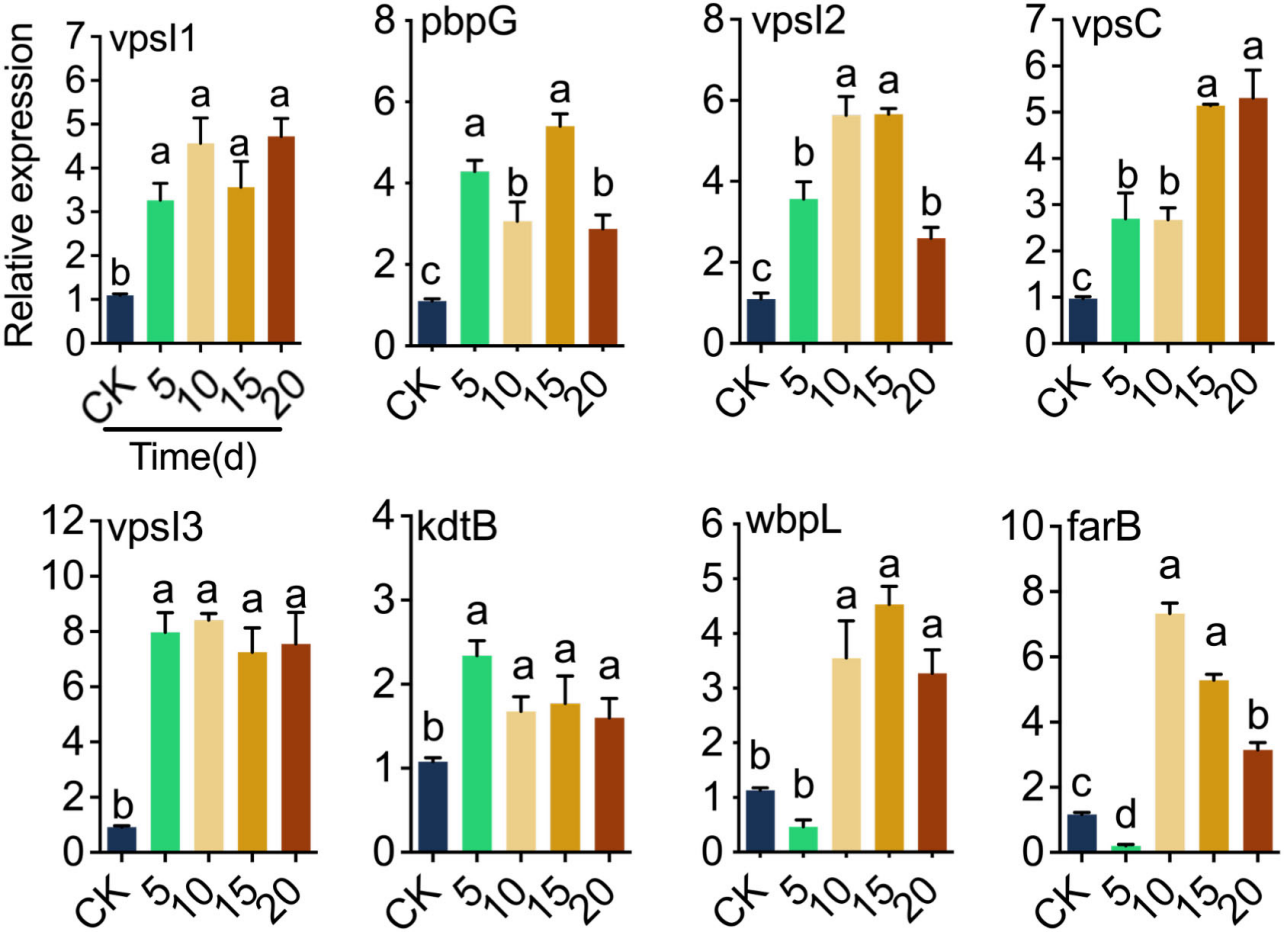
Genes specifically contributing to tomato rhizosphere soil microorganism colonization. Colonization-related gene expression at different times (0, 5, 10, 15, and 20 days) after inoculation with LP0308 (10^7^ CFU/ml) by RT-qPCR. The mean ± standard error of the mean (SEM) of experiments performed in quintuplicate, with five tomato plants/experiment, are shown. Variation analysis was performed by one-way ANOVA followed by Tukey’s *post-hoc* test. Different letters represent significant differences at *P* < 0.05. Encoding biofilm (*vpsI*1, *vpsI*2, *vps*C, and *vpsI*3), immune modulation (*pbpG*, *kdtB*, and *wbpL*) and antimicrobial activity (*farB*) proteins.

Quantitative real-time PCR was utilized to determine how the response of LP0308 gene expression after colonization in soil on tomato plant. The expression of genes encoding biofilm (vpsI1, vpsI2, vpsC, and vpsI3), immune modulation (pbpG, kdtB, and wbpL), and antimicrobial activity (farB) proteins was measured based on their colonization-related functions (Figure 6). After LP0308 successfully colonized the rhizosphere soil, three biofilm-related, three immune modulation-related, and one antimicrobial activity-related showed substantially increased expression (Figure 6). In particular, compared with the level observed at 0 day, the expression of pbpG (fivefold), vpsI3 (eightfold), and farB (sevenfold) reached extremely significant levels at 15 days (*t* = 14.08, df = 8, *P* < 0.0001), 10 days (*t* = 29.66, df = 8, *P* < 0.0001), and 10 days (*t* = 19.41, df = 8, *P* < 0.0001), respectively (Figure 6).

### Growth-promoting effects of LP0308 on tomato buds and seedlings

**FIGURE 7:**
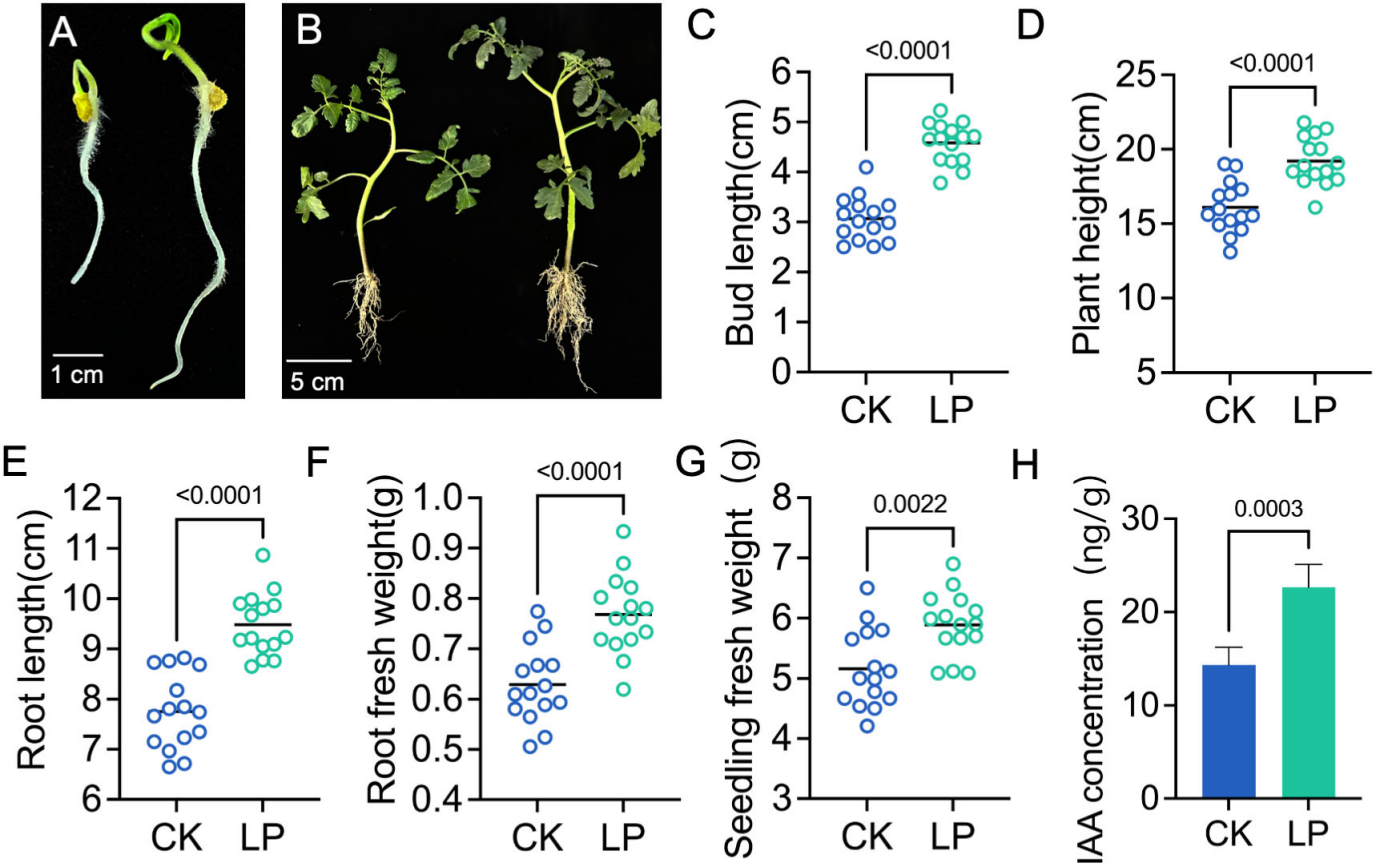
Effects of LP0308 on the growth and development of tomato. The tomato seedlings were treated with either the control or different concentrations of LP0308 (1 × 10^7^ CFU/ml) and sampled after 20 days. The promoting effect of LP0308 on the germination of tomato seeds **(A)** and seedlings **(B)**. The following parameters were measured: **(C)** bud length, **(D)** plant height and **(E)** root length, **(F)** root fresh weight, and **(G)** seedling fresh weight. **(H)** The contents of indoleacetic acid (IAA) of tomato root. Data were analyzed from 15 seedlings for each treatment. The error bars indicate the ±SDs of the means. Statistical significance was determined by two-sided Student’s *t*-test.

Tomato seed treatment with LP0308 increased the seed germination and seedling growth compared to the control treatments (Figures 7A, B). Moreover, LP0308 significantly increased bud length (Figure 7C), plant height (Figure 7D), primary root length (Figure 7E), root fresh weight (Figure 7F), and whole-seedling fresh weight (Figure 7G) (*P* < 0.05; *n* = 15). For example, primary root length was 4.58 ± 0.41 cm in LP0308-treated seedlings vs. 3.07 ± 0.45 cm in the control (CK) group (*t* = 6.866, df = 28, *P* < 0.0001). In addition, root indole-3-acetic acid (IAA) concentration rose from 14.35 ± 1.90 to 22.67 ± 2.44 ng/g, representing a 1.58-fold increase (*t* = 6.029, df = 28, *P* = 0.0003) (Figure 7H).

### Effect of LP0308 on soil properties and soil enzyme activities

**FIGURE 8:**
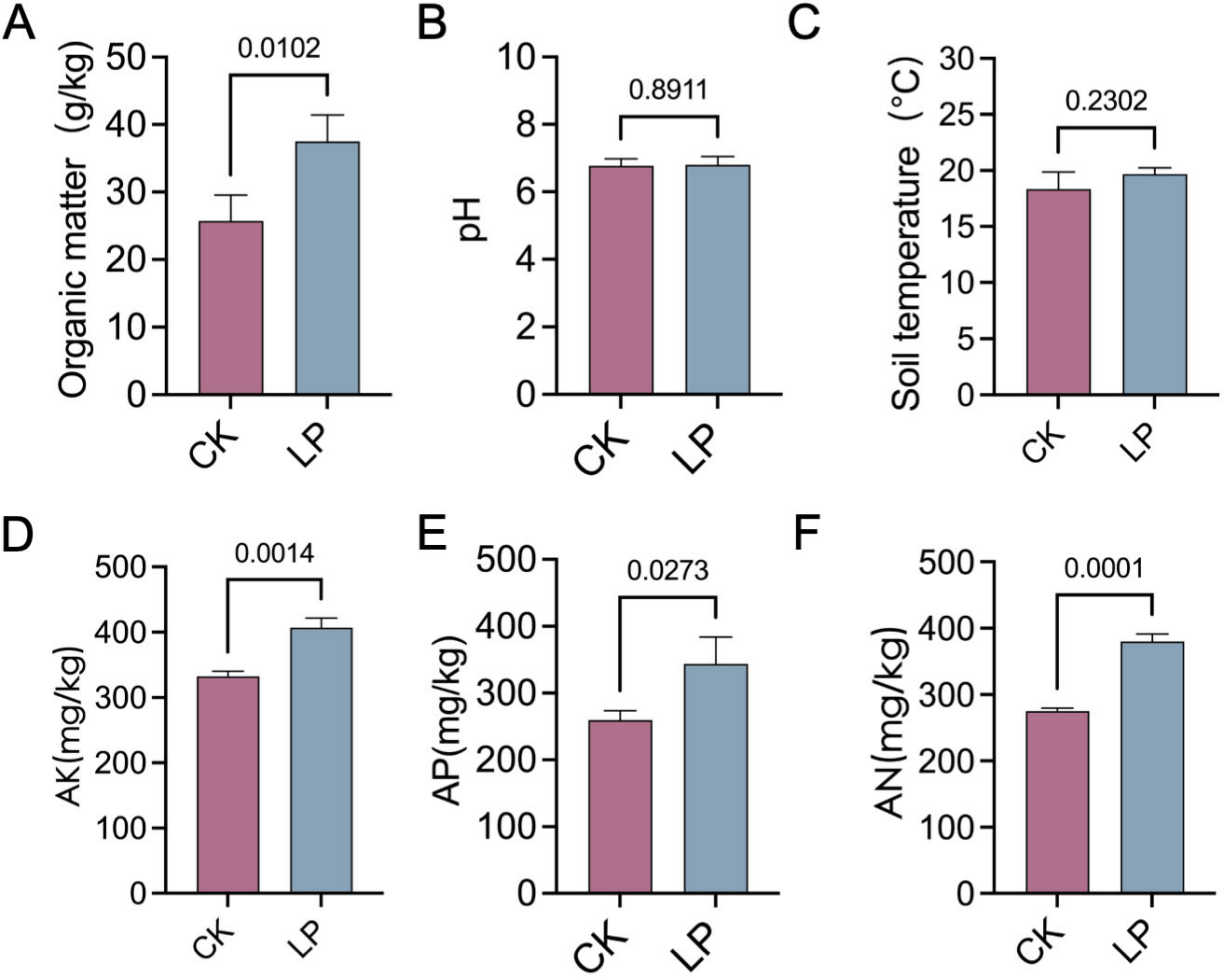
Effects of LP0308 on rhizosphere soil physicochemical properties. Soil from tomato rhizospheres was analyzed after treatment with LP0308 (LP) or sterile control (CK). **(A)** Organic matter, **(B)** soil pH, **(C)** soil temperature, **(D)** available potassium (AK), **(E)** available phosphorus (AP), and **(F)** available nitrogen (AN). Bars represent means ± SD (*n* = 15). *P* values derive from two-tailed Student’s *t*-tests comparing LP with CK for each parameter.

**FIGURE 9:**
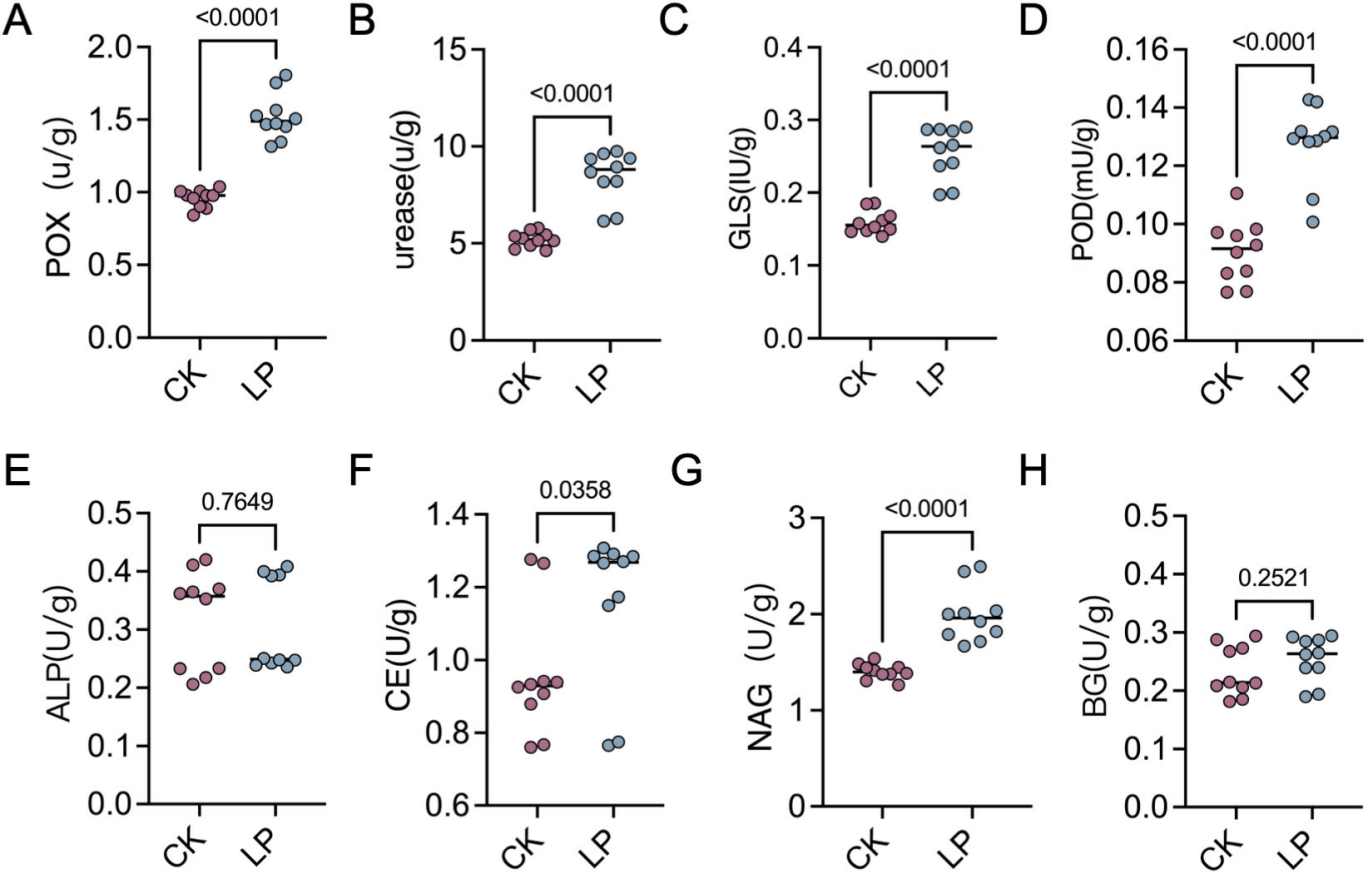
Effects of LP0308 inoculation on rhizosphere soil enzyme activities. Activities of eight functionally diverse enzymes were quantified in tomato rhizosphere soils following LP0308 (LP) or control (CK) treatment: polyphenol oxidase (POX, **A**), urease **(B)**, β-glucosidase (GLS, **C**), peroxidase (POD, **D**), alkaline phosphatase (ALP, **E**), cellulase (CEU, **F**), N-acetyl-β-D-glucosaminidase (NAG, **G**), and β-glucosidase (BG, **H**). Points represent individual soil samples; bars (or horizontal lines) denote means ± SD (*n* = 10). *P* values are from two-tailed Student’s *t*-tests comparing LP with CK for each enzyme.

LP0308 treatment led to a pronounced enrichment of soil OM (37.52 ± 3.91 vs. 25.72 ± 3.82) and AK (407.33 ± 14.47 vs. 332.33 ± 8.02), AP (343.66 ± 40.41 vs. 259.67 ± 14.15), and AN (380.22 ± 4.55 vs. 275.30 ± 4.55) compared with the control group (*P* < 0.05). However, soil pH (6.80 ± 0.25 vs. 6.77 ± 0.20) and soil temperature (19.69 ± 0.58 vs. 18.33 ± 1.53) were statistically indistinguishable from the control (*P* > 0.05) (Figures 8A–F). Concurrently, enzymatic profiling revealed significant upregulation of POX (1.48 ± 0.13 vs. 0.95 ± 0.06), urease (8.48 ± 1.38 vs. 5.20 ± 0.42), GLS (0.25 ± 0.04 vs. 0.16 ± 0.01), POD (0.13 ± 0.01 vs. 0.09 ± 0.01), CE (1.20 ± 0.17 vs. 0.97 ± 0.19), and NAD (2.01 ± 0.29 vs. 1.39 ± 0.07) activities under LP0308 inoculation (*P* < 0.05) compared with the control group (*P* < 0.05), whereas ALP (0.31 ± 0.08 vs. 0.33 ± 0.08) and BG (0.25 ± 0.04 vs. 0.23 ± 0.04) did not differ statistically from the control (*P* = 0.76 and 0.25, respectively) (Figures 9A–H).

## Discussion

The successful colonization of the in soil on tomato plant by probiotics is an essential step in controlling beneficial microbe ecosystems and plant health or soil fertility. In this study, LP0308 was introduced into the soil of tomato, and it could stably persist and proliferate. LP0308 levels in the rhizosphere soil significantly increased and demonstrated long-term stability during this period (0–25 days). This colonization efficiency was considerably stronger than the effect reported by Qian et al. (2022), who found that the density of strain *Hansschlegelia zhihuaiae* S113 colonizing rhizosphere soil reached maximum level (4.7 × 10^8^ cells/g) on the 5th day, but the colonization effect of strain S113 gradually weakened after the 10th day. The high survival rate of LP0308 in soil means the *L. plantarum* might be used as probiotics. *L. plantarum* strain LP0308 was confirmed as a possible plant PGPR, which significantly promoted bud length, plant height, primary root length, root fresh weight, and whole-seedling fresh weight. According to Amprayna et al. (2016), LAB exhibit PGP characteristics, as they can produce the coenzyme IAA and solubilize minerals. LAB colonizing the plant rhizosphere can enhance the plant’s biological traits and boost yield. For instance, rice seeds coated with *Lactococcus lactis* significantly increased root and shoot lengths (Amprayna et al., 2016). Moreover, *L. lactis* notably promoted both growth and yield in cabbage (Somers et al., 2007). However, soil sterilization inherently alters physicochemical gradients and excludes biotic interactions, which may overestimate strain persistence; therefore, future work will assess LP0308’s colonization and resilience in natural, non-sterile soils to confirm its agronomic relevance.

In addition, we found that LP0308 altered the microbial composition of the rhizosphere soil and significantly increased the abundance of *Bacillus*. LAB are widespread microorganisms that hold promising benefits for enhancing crop and livestock production (Ikeda et al., 2013). Together with LAB, other Firmicutes (e.g., *Bacillus* species) are ubiquitous, often found as soil inhabitants and easily transferred from soil to roots (Arkhipova and Anokhina, 2009). Studies have found that some strains of *Bacillus* can inhibit the pathogenic soil-borne fungus *Fusarium graminearum* and produce cytokinins, acting as PGP probiotics (Arkhipova and Anokhina, 2009; Zhao et al., 2014). Wang et al. (2019) found that *Bacillus stearothermophilus* increases the relative abundance of LAB strains in the soil and exhibits antagonistic effects against plant pathogens in the soil. Similar studies have found that *Pediococcus pentosaceus* positively correlated with *L. plantarum* (Jiang et al., 2021). Recently, LAB and other *Bacillus*-based biofertilizers have been successfully integrated with established microorganisms in environmental and agriculture applications. These microbial biofertilizers are known to boost crop yields, facilitate the replenishment of essential minerals in plant roots, and improve the metabolic processes involved in OM decomposition (Khoso et al., 2024).

Interestingly, LP0308 significantly reduced potentially pathogenic and promoted biofilm formation of rhizosphere soil microorganism. We found that the cell-free supernatant of LP0308 significantly inhibited *R. solanacearum* and *F. oxysporum*. PGPR are known to produce secondary metabolites antagonistic to various soil-borne pathogens (Uwaremwe et al., 2021). These results support previous research that shows that *L. plantarum* significantly reduces the virulence factors, biosurfactants formation of pathogenic bacteria such as *Pseudomonas aeruginosa*, *Staphylococcus aureus*, and *Escherichia coli* (Anupama et al., 2009a; Ahn et al., 2018). Research by Anupama et al. (2009b) revealed that *L. plantarum* exhibits antagonistic activity against plant pathogenic bacteria *Xanthomonas campestris* and *R. solanacearum* (Visser et al., 1984). Further studies have shown that LAB eradicate the formation of pathogenic microorganism surface adhesion by producing glycolipid biosurfactants (Manjarres Melo et al., 2021; Guan et al., 2023). *L. plantarum* isolated from traditional sourdoughs has shown significant antagonism activity against *Erwinia amylovora*, *Penicillium digitatum*, and *B. cinerea in vitro* (Tremonte et al., 2017; Sadanov et al., 2023). In addition, *Lactobacillus buchneri* 6M1 and *Lactobacillus brevis* 5M2 isolated from corn silages showed antifungal activity against *F. graminearum* and carboxylesterase activities (Paradhipta et al., 2021). The anti-pathogenic microorganism activity of LAB is typically expressed through competition for essential nutrients and the production of bioactive substances such as organic acids, carbon dioxide, ethanol, amines, hydrogen peroxide, fatty acids, acetaldehyde, diacetyl, cyclic dipeptides, vitamin, exopolysaccharides bacteriocins, or bacteriocin-like inhibitory substances (Rouse et al., 2008; Arena et al., 2019). A key mechanism for probiotic colonization is the formation of biofilms, enabling survival under stress conditions in the host, while facilitating nutrient exchange and coordinating host activities (Rasooly et al., 2022). In this study, LP0308 may enhance its competitive advantage in the rhizosphere microbiome through biofilm formation. However, reliance on *in vitro* assays and functional predictions means that the antagonistic effects and biofilm-mediated competitive advantage remain unvalidated under the rhizosphere’s complex biotic and abiotic conditions.

The successful colonization of LP0308 led to drastically increased expression of encoding biofilm-associated gene (*vpsI*1, *vpsI*2, *vpsC*, and *vpsI*3), immune modulation (*pbpG*, *kdtB*, and *wbpL*) and antimicrobial activity (*farB*) in the rhizosphere soil on a genome-wide scale. For example, *Vibrio cholerae* enhances the production of exopolysaccharide (*EPS*) by regulating the transcription of *VPS* genes on its chromosome, thereby enabling the formation of well-developed, mature biofilms that improve its resistance to environmental stress (Casper-Lindley and Yildiz, 2004; Butz et al., 2021). Penicillin-binding proteins (*PBPs*), which are serine-based enzymes, act as redox sensors to regulate cell wall synthesis, as well as nutrient-driven cell growth, division, and proliferation, and are essential for plant growth (Sacco et al., 2022). Another study showed that the *farB* gene is involved in maintaining cell membrane integrity, regulating intracellular fatty acid levels, and contributing to bacterial resistance, particularly against antimicrobial long-chain fatty acids (Yin et al., 2019; Khoder et al., 2022). Genes involved in core enterococcal and genome plasticity are key drivers of rhizosphere soil colonization.

Additionally, application of LP0308 markedly improved soil nutrient availability and stimulated key enzymatic activities, indicating its potential to enhance rhizosphere health and fertility. In this study, OM content increased from 25.72 g/kg in the control to 37.52 g/kg following LP0308 treatment. Higher OM could facilitate the growth and physiological activity of plant roots, as well as the metabolism of microorganisms, thus promoting enzymatic secretion by plants and microbes (Fanin et al., 2014). Lee et al. (2022) found that soil inoculation with *Rhodopseudomonas palustris* PS3 enhanced both soil nutrient availability and tomato endogenous nitrogen content, raising soil nutrient availability from 35% to 56% in an organic field. In addition to nutrients, we also found that the activities of some soil enzymes, such as POX, urease and ALP, were significantly increased. Since soil enzyme activity is regarded as an indicator of microbial activity (Xiao et al., 2020). We hypothesize that LP0308 inoculation can stimulate microbial activity in the soil. Research found that *Sinorhizobium meliloti* CCNWSX0020 application improved soil microbial parameters, such as soil enzyme activity (i.e., β-glucosidase activity and ALP) and microbial biomass, thereby promoting alfalfa growth (Duan et al., 2021). For example, phosphatases play a critical role in catalyzing the hydrolysis of organic phosphorus compounds and are essential for enhancing overall soil quality (Yang et al., 2017; Wang et al., 2020). Therefore, we presume that LP0308 not only proliferates within the soil microbiome but also activates native microbial functional guilds, thereby accelerating nutrient turnover and availability.

## Conclusion

The conducted study allowed a holistic assessment of the LP0308 colonization strategy and function in the tomato rhizosphere soil. Through a deeper understanding and utilization of *L. plantarum*, we can better address challenges in agriculture and environmental sectors, paving the way for a more sustainable future. These microorganisms offer a means of working synergistically with nature, contributing to soil improvement, plant protection, and the enhancement of environmental quality. The findings in our study provide evidence of the fundamental basis of rhizosphere colonization and signify an important step toward the development of robust bioinoculants in sustainable agriculture.

## Data Availability

The datasets presented in this study can be found in online repositories. The names of the repository/repositories and accession number(s) can be found in the article/Supplementary material.
