# Supplementary material for: Features and rhizosphere colonization strategies of Lactobacillus plantarum 0308 in soil-tomato systems
Source: Front Microbiol. 2025 Aug 29;16:1652881. doi: 10.3389/fmicb.2025.1652881 (PMC12426192; doi:10.3389/fmicb.2025.1652881)
Supplement: Supplementary file 1 [file Data_Sheet_1.docx]

Supplementary Material

**Separation and purification of LP0308**

A culture of LP0308 was established by inoculating a single colony from the activated culture plate into 250 mL of MRS broth, followed by incubation in an orbital shaker at 180 rpm and 37°C for 48 hours. Molecular analysis was conducted by isolating genomic DNA and amplifying the bacterial 16S rRNA gene using the universal primers 27 F and 1492R. The PCR reactions were conducted using the following program: 3 min of denaturation at 95°C, 27 cycles in the first step and 13 cycles in the second step of 30 s at 95°C, 30 s for annealing at 55°C, and 45 s for elongation at 72°C, and a final extension at 72°C for 10 min. The resulted PCR products were extracted from a 2% agarose gel and further purified using the AxyPrep DNA Gel Extraction Kit (Axygen Biosciences, Union City, CA, USA) and quantified using QuantiFluor™-ST (Promega, USA) according to the manufacturer’s protocol. The PCR products were subsequently synthesized and sequenced by Sangon Biotech (Shanghai, China).

**Community assembly processes of LP0308 in Rhizosphere soil**

To evaluate community assembly processes at each site, we calculated the nearest taxon index (βNTI) and Bray–Curtis-based Raup–Crick (RCBray) for null model analysis as per Stegen et al. (Stegen et al., 2012; Stegen et al., 2013). Classified community pairs into underlying drivers of species selection, dispersal limitation, homogeneous dispersal, and drift based on βNTI and RCBray. Aligned sequences of representative ASVs were used to construct a maximum-likelihood tree in FastTree (Price et al., 2010). The null model expectation was generated using 999 randomizations in the “Picante” R package. A significant deviation (i.e., |βNTI| > 2) indicates the dominance of selection processes (Selection). βNTI values > +2 indicate significantly more than expected phylogenetic turnover, whereas βNTI values < −2 indicate significantly less than expected phylogenetic turnover; the relative contributions of dispersal limitation for spatial turnover in community composition were estimated as the fraction of pairwise comparisons with |βNTI| < 2; dispersal limitation was quantified as the fraction of pairwise comparisons with |βNTI| < 2 and RCBray > 0.95; the relative influence of homogenizing dispersal was quantified as the fraction of pairwise comparisons with |βNTI| < 2 and RCBray < –0.95. Lastly, if |βNTI| < 2 and |RCbray| < 0.95, then weak selection, weak dispersal, diversification, and/or drift. This condition has been referred to as “undominated” assembly (Stegen et al., 2015; Zhou et al., 2017; Tripathi et al., 2018).

**Germination of Tomato Seeds**

The specific steps are as follows: The LP0308 strain was streaked onto MRS solid medium, and single colonies were transferred to MRS liquid medium for 24 hours of shaking culture (30°C, 180 rpm). The cultured bacterial suspension was centrifuged at 10,000 g for 5 min. The supernatant was then filtered through a 0.22 μm filter (Millipore, Germany), and tomato seeds were soaked in the filtrate for 30 minutes (LP0308 group). As a control, seeds were soaked in sterile MRS liquid medium (CK group). They were placed on moist and sterile filter paper and incubated at 27°C for 5–6 days in Petri dishes. After the seeds germinate, transfer them to a sterile glass bottle containing 1/2 Murashige and Skoog (MS) medium, supplemented with 1% (w/v) sucrose and 0.8% (w/v) agar and growth for 7 days.

**Supplementary Tables**

**Supplementary** **Table S1.** The following primers were used in this study.

| Primer | F | R |
| --- | --- | --- |
| LP  BA  vpsl1  vpsI1  pbpG  vpsI2  vpsC  vpsI3  kdtB  wbpL  farB | TCACATTACCATACGGTTAA  CTGTATTTAGTTGAACGGG  ACTAAAGTTTTGCATACTGA  TCACATTACCATACGGTTAA  CTGTATTTAGTTGAACGGG  ACTAAAGTTTTGCATACTGA  TATTGCAGGCTATGTTTGCC  AGCAATCTACACGTTAAGGA  GTCGATTAGTTGACCGGTTG  GATGGGTTGGACGGATTAGT  GGACCATTAATCGGACCGGT | GTGAATTTTACGAAATGACT  ATGCGAGTTTTATTAGCGCT  GGCGAGACCGTTATTGAGCA  GTGAATTTTACGAAATGACT  ATGCGAGTTTTATTAGCGCT  GGCGAGACCGTTATTGAGCA  TCACAATCGAGGATGGCTGT  TACTAGCGCCCATCCTGCTC  TCGAAACGATTTGCTTGATT  GTCATTGAAACGGCTTCAGT  CAAGTATTGAGATGTTAATTC |

**
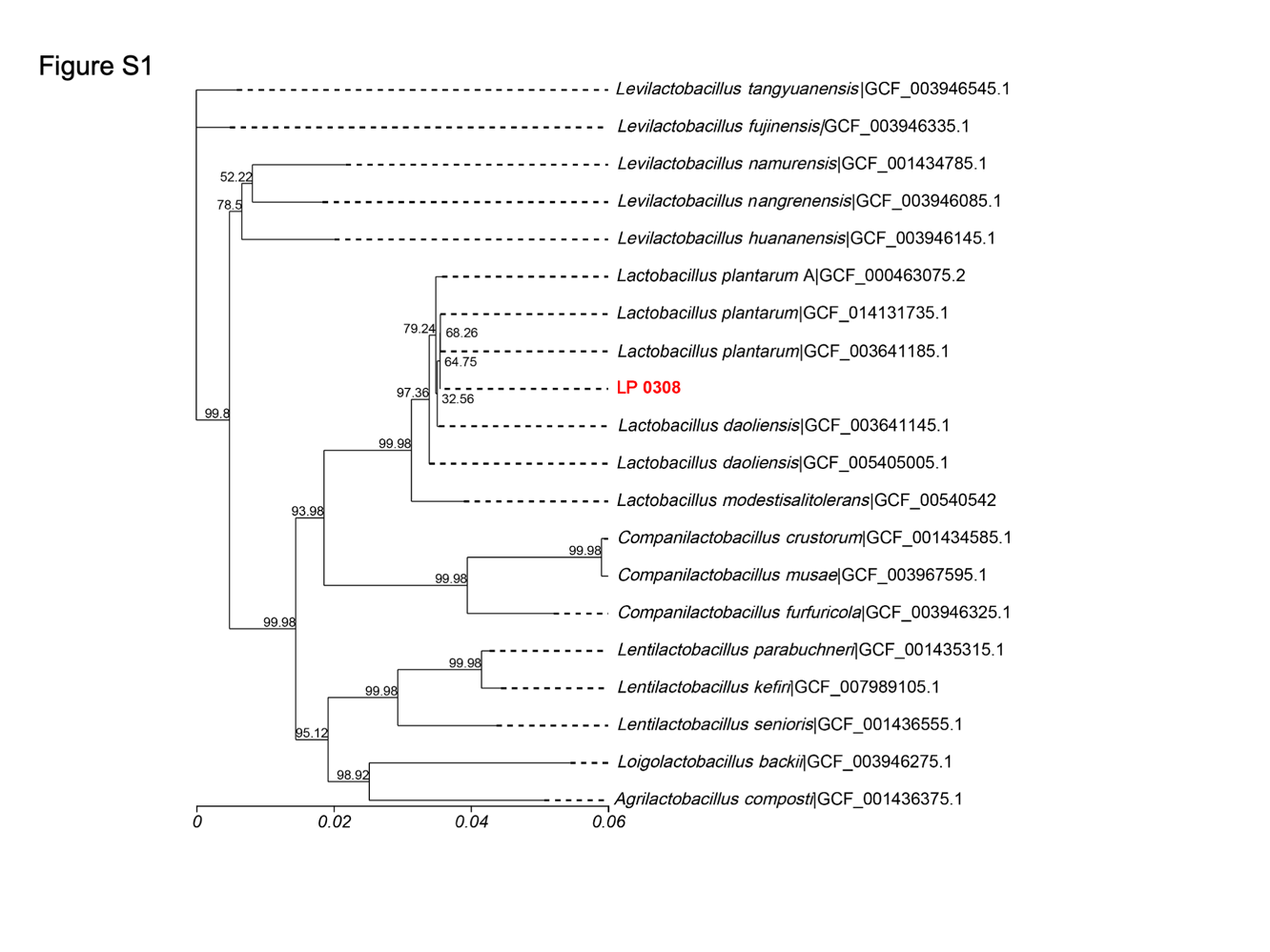
Supplementary Figures**

**Supplementary Figure S1.** Phylogenetic tree indicating the relationship of *L. plantarum* LP0308 strain 16S rRNA gene sequences compared with sequences of related bacterial species from GenBank.

*
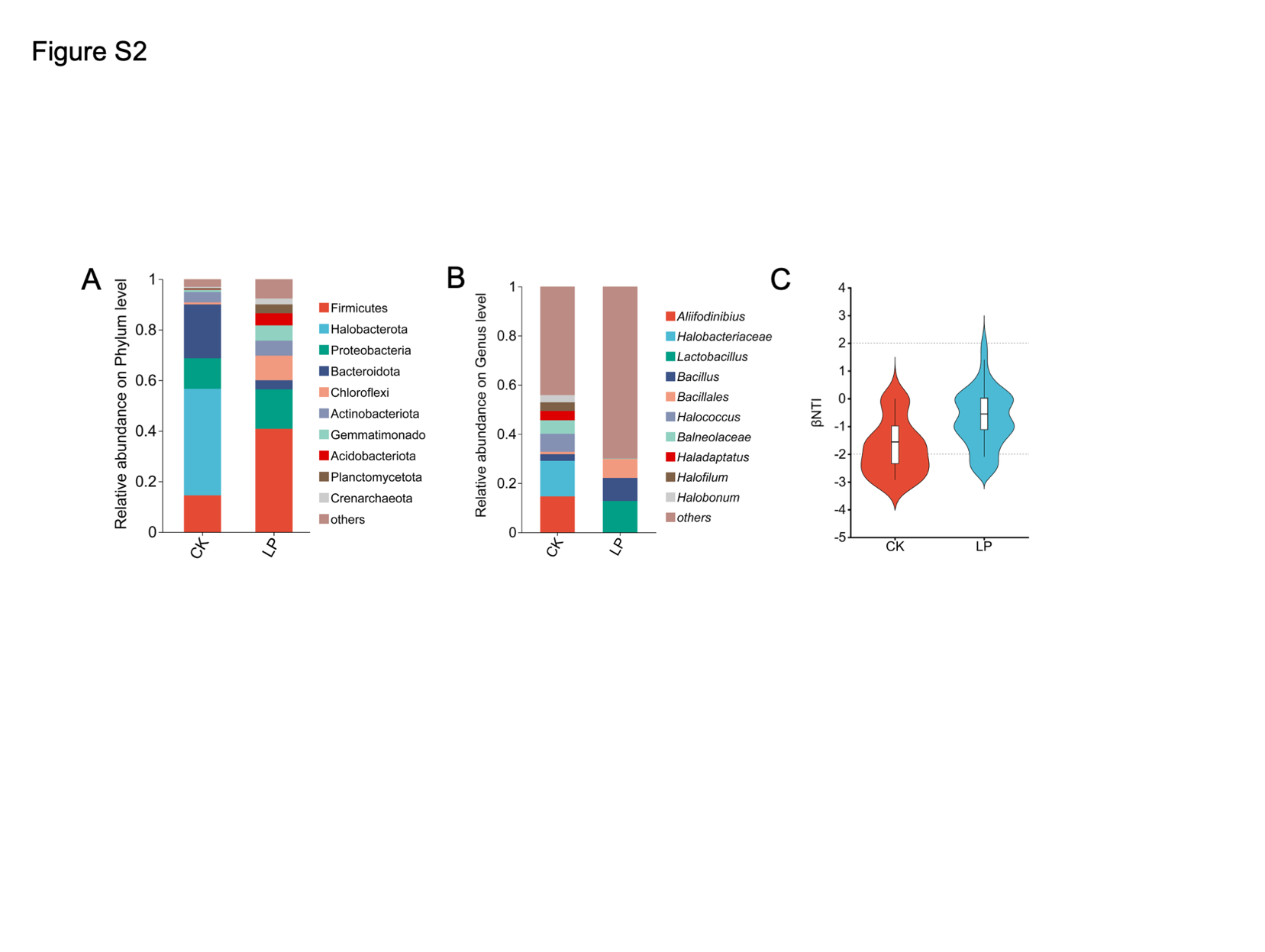
*

**Supplementary Figure S2.** Relative abundance of bacterial phyla(A) and genus(B) in different samples. Taxa with abundances < 1% are included in “others”. The βNTI distributions of bacterial(C) communities across soil niches mediated by *L. plantarum* 0308. Horizontal gray dashed lines indicate upper and lower significance thresholds at βNTI = +1.96 and -1.96, respectively.

**
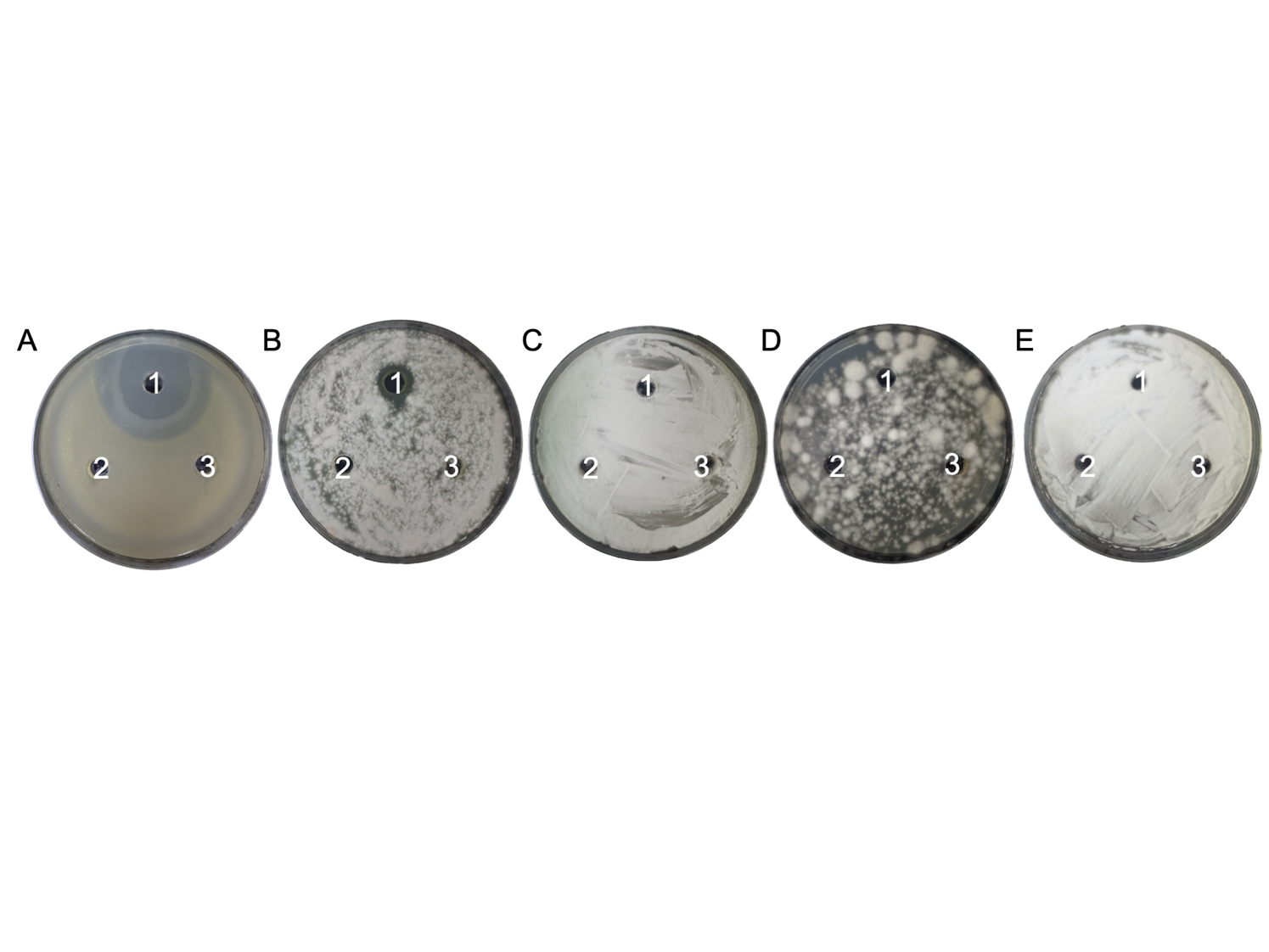
**

**Supplementary Figure S3**. Characterization of LP0308 inhibitory activity. Typical results of agar diffusion assays using *R. solanacearum* (A), *F. oxysporum* (B), *C. fulvum* (C), *A. solani* (D), and *B. cinerea* (E) as the indicator. (1) cell-free culture supernatant of LP0308; sterile water (2) and medium (3) was used as a negative control.

**REFERENCES**

Price, M. N., Dehal, P. S., and Arkin, A. P. (2010). FastTree 2 – Approximately Maximum-Likelihood Trees for Large Alignments. *PLOS ONE.* 5, e9490. doi:10.1371/journal.pone.0009490.

Stegen, J. C., Lin, X., Konopka, A. E., and Fredrickson, J. K. (2012). Stochastic and deterministic assembly processes in subsurface microbial communities. *The ISME journal.* 6, 1653-1664. doi:10.1038/ismej.2012.22.

Stegen, J. C., Lin, X., Fredrickson, J. K., and Konopka, A. E. (2015). Estimating and mapping ecological processes influencing microbial community assembly. *Frontiers in Microbiology.* 6. doi:10.3389/fmicb.2015.00370.

Stegen, J. C., Lin, X., Fredrickson, J. K., Chen, X., Kennedy, D. W., Murray, C. J. et al. (2013). Quantifying community assembly processes and identifying features that impose them. *The ISME journal.* 7, 2069-2079. doi:10.1038/ismej.2013.93.

Tripathi, B. M., Stegen, J. C., Kim, M., Dong, K., Adams, J. M., and Lee, Y. K. (2018). Soil pH mediates the balance between stochastic and deterministic assembly of bacteria. *The ISME Journal.* 12, 1072-1083. doi:10.1038/s41396-018-0082-4.

Zhou, J., and Ning, D. (2017). Stochastic Community Assembly: Does It Matter in Microbial Ecology? *Microbiology and Molecular Biology Reviews.* 81, e00002-00017. doi:10.1128/MMBR.00002-17.
